# ASO Author Reflections: Collaborative Surgical Care in the Transgender Community

**DOI:** 10.1245/s10434-024-16095-x

**Published:** 2024-08-21

**Authors:** Rachel L. McCaffrey, Andrew J. James

**Affiliations:** 1 Division of Surgical Oncology and Endocrine Surgery, Department of Surgery, Vanderbilt University Medical Center, Nashville, TN USA; 2 Department of Plastic Surgery, Vanderbilt University Medical Center, Nashville, TN USA

## Past

As the nation grapples with the opinions and politics of those in the transgender community, the medical community must focus on collaborative care for these individuals. As part of the queer community, my focus through the lens of breast surgical oncology, is the risk of breast cancer in transgender and nonbinary patients. This is a call to investigate the risk of breast cancer development in the transmasculine and nonbinary population to further individualize transgender breast cancer screening, improve collaborative surgical care, and possibility elucidate new treatment pathways for breast cancer patients overall.^1,2^

## Present

We performed a single-institution review of the largest known retrospectively collected cohort of transgender and nonbinary patients undergoing chest masculinization surgery (i.e., top surgery). Of the 865 patients included for review, high-risk or malignant findings were noted in 12 of 1730 breast specimens, only 2 of which were malignant. All malignant lesions and 50% of proliferative lesions with atypia were found in patients aged 35 years or older. Additionally, patients younger than 25 years were 70% less likely to have any incidental finding on pathological evaluation (odds ratio [OR] 0.3, *p *< 0.01, 95% confidence interval [CI] 0.18–0.50).^3^ Within our practice, informed by this data, we assess all patients for breast cancer risk and screen any patient older than 40 years or at high risk of breast cancer with imaging before chest masculinization surgery to individualize surgical care.

## Future

As more patients undergo chest masculinization surgeries, there is a need for surgical oncology collaboration in transgender surgical programs. Measures should include screening for breast cancer risk (Breast Cancer Risk Assessment Tool (BCRAT), Tyrer-Cuzick Risk calculator, etc.), preoperative breast cancer screening for those older than 40 or at high risk for breast cancer, and collaboration with a breast surgical oncologist for those patients who may benefit from a more risk-reductive surgery or, rarely, upfront oncologic care.^4^ Collaborative programs with these measures will, without a doubt, improve the care of transmasculine and nonbinary patients.
